# Medical image fusion based on machine learning for health diagnosis and monitoring of colorectal cancer

**DOI:** 10.1186/s12880-024-01207-6

**Published:** 2024-01-24

**Authors:** Yifeng Peng, Haijun Deng

**Affiliations:** 1 Department of General Surgery, Southern University of Science and Technology Hospital, Shenzhen, 518055 Guangdong China; 2 Department of General Surgery, Nanfang Hospital, Southern Medical University, Guangzhou, 510515 Guangdong China

**Keywords:** Colorectal Cancer Diagnosis, Medical Image Fusion, Computational Intelligence Systems, Safety Medicine, Multimodal Medicine

## Abstract

With the rapid development of medical imaging technology and computer technology, the medical imaging artificial intelligence of computer-aided diagnosis based on machine learning has become an important part of modern medical diagnosis. With the application of medical image security technology, people realize that the difficulty of its development is the inherent defect of advanced image processing technology. This paper introduces the background of colorectal cancer diagnosis and monitoring, and then carries out academic research on the medical imaging artificial intelligence of colorectal cancer diagnosis and monitoring and machine learning, and finally summarizes it with the advanced computational intelligence system for the application of safe medical imaging.In the experimental part, this paper wants to carry out the staging preparation stage. It was concluded that the staging preparation stage of group Y was higher than that of group X and the difference was statistically significant. Then the overall accuracy rate of multimodal medical image fusion was 69.5% through pathological staging comparison. Finally, the diagnostic rate, the number of patients with effective treatment and satisfaction were analyzed. Finally, the average diagnostic rate of the new diagnosis method was 8.75% higher than that of the traditional diagnosis method. With the development of computer science and technology, the application field was expanding constantly. Computer aided diagnosis technology combining computer and medical images has become a research hotspot.

## Introduction

At present, rectal digital embolism, barium enema and other examination methods can not detect the intestinal wall and tumor metastasis of patients in time, nor can the growth of intestinal cavity be observed. The preoperative lymph node metastasis and local diffusion of rectal cancer can be effectively evaluated according to imaging examination, and whether it can be surgically removed can be determined by combining imaging examination. Colorectal cancer is a common malignant tumor in the digestive tract. The incidence rate and mortality of colorectal cancer are on the rise worldwide. Early diagnosis and treatment are key factors affecting patient prognosis. Since its inception, artificial intelligence (AI) has been continuously studied and has made significant progress in recent years [1]. In the field of medicine, the application of artificial intelligence in endoscopy, medical imaging, and pathology can provide reliable reference opinions for doctors, reduce differences in experience between doctors, and help doctors make more accurate diagnostic decisions.

Many scholars have studied the diagnosis and monitoring of colorectal cancer. Ma Zhi Yao believed that with the increasing incidence and mortality of colorectal cancer, an early and accurate diagnosis is considered a primary condition for prevention and treatment [2]. Shaukat Aasma believed that research is currently underway on the diagnostic accuracy and longitudinal performance of blood tests, which may undermine the prospects of CRC screening. The selection based on imaging, including colon capsule, magnetic resonance colonography, and CT capsule, is also being actively tested in research [3]. Patel, Swati G focused on updating the 2017 colorectal cancer screening recommendations of the American Cancer Multisocial Working Group, which represents the American Society of Gastroenterology, the American Society of Gastroenterology, and the American Society of Gastroenteroscopy [4]. Campos-da-Paz Mariana found through research that early carcinoembryonic antigen is a recommended prognostic indicator in the early diagnosis and monitoring of colorectal cancer. The high level of CEA (Carcinoembryonic Antigen) is closely related to the development of CRC, and the increase of its markers is expected to decrease after surgical treatment [5]. Ji Jianguang believed that colorectal cancer is the most common malignant tumor and the most common cancer in the world, and the development of new biomarkers is an important public health strategy [6]. Li Huizi believed that colorectal cancer is a common cancer, and its etiology is caused by genes and epigenes [7]. Brouwer Nelleke PM’s research found that in the diagnosis and treatment of colorectal cancer, its long-term development trend must be evaluated, so as to provide more research and innovation for the health management of cancer patients [8]. The above research has achieved good results, but with the continuous updating of technology, there are still some problems.

Machine learning medical imaging AI has been applied much in the therapeutic process. Li Xiaoxiao introduced Laplace decision graph decomposition methods to obtain complementary information, redundant information, and low-frequency subband images [9]. Tawfik Nahed believed that the fusion of multimodal medical images is to merge multiple images from one or more images [10]. Azam, Muhammad Adeel briefly reviewed the evaluation and statistical results of different medical imaging modes and related multimodal databases. The medical imaging mode is organized based on radiation, visible light imaging, microscopy, and multimodal imaging [11]. Yadav Satya Prakash believed that an efficient image fusion technology can maintain all the useful and significant information collected from the original image without introducing any defects or meaningless distortion [12]. Tirupal T believed that multimodal medical image fusion is a research that combines several images from different sources into one image to obtain better information [13]. Arif Muhammad analyzed many medical imaging scholars trying to extract additional images and relevant information from different medical images to create a fused medical image [14]. Wang Lifang considered multimodal medical image fusion as an effective diagnostic tool. However, image fusion algorithms based on multi-scale decomposition have problems of low contrast and energy loss [15]. Luo Yueyuan used endogenous contrast agents such as melanin and hemoglobin to monitor the concentration of substances related to tumor angiogenesis in real-time and non-invasively, or provided information about tumor structure and its molecules by combining molecular targeted exogenous contrast agents with antibodies or peptides, thus realizing morphological and functional imaging [16]. The above research shows that the application of machine learning-based medical imaging AI has a positive effect, but there are still some problems.

The surgical cure rate and survival rate of colorectal cancer have been fluctuating for many years, and the reason for treatment failure is the high local recurrence rate. Therefore, comprehensive treatment is needed to improve the efficacy of colorectal cancer. The innovation of this article is as follows: 1) this article proposes a large fusion criterion based on regional variance method. This criterion not only considers the correlation between adjacent pixels, but also preserves the details of the original image as much as possible to ensure the clarity of the image. 2) Low frequency coefficients are used to represent the main information of an image. The use of neighborhood averaging method to obtain fused low-frequency coefficients can effectively improve the contrast and brightness of the image.

## Application of medical image fusion

### Medical image technology

**Fig. 1:**
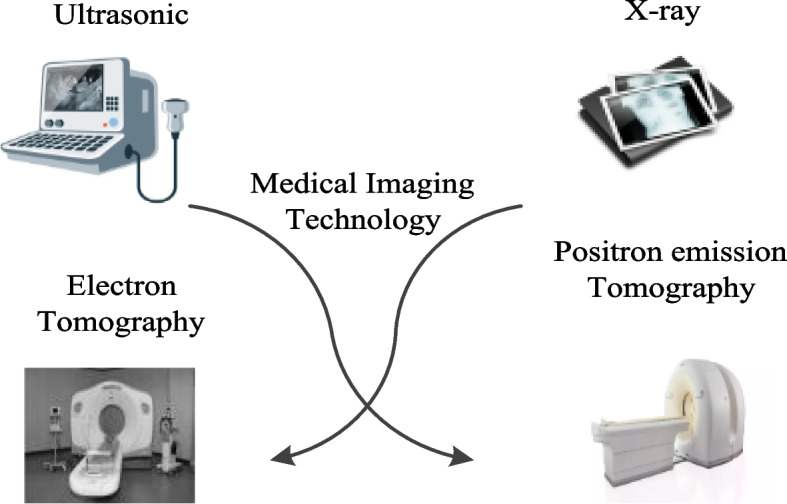
Application of multimodal security medical image fusion

Medical imaging technology provides rich clinical information, such as ultrasound, X-ray, electronic tomography, MRI, DSA, positron emission tomography, etc. Medical imaging can provide different information for different organs and tissues of the body, such as CT (Computed Tomography) and MRI, while thermoplastic polyester and single-photon emission computed tomography can provide corresponding functional information, as shown in Fig. 1 [17, 18]:

In practice, single mode images often cannot provide necessary information for doctors. In order to make accurate diagnosis and develop appropriate treatment plans, multiple mode images should be combined to obtain more information, so as to better understand the overall condition of the disease. Multimodal images mainly include RGB images, infrared, near-infrared and other multispectral images, depth maps, and various medical images. For RGB images, flash / no flash images, multi focus images, multi exposure images, etc., are generally considered multimodal. These can all play an important role in developing appropriate treatment plans. For example, CT can combine bones, soft tissues and blood vessels well through X-ray absorption coefficients of different tissues and organs and CT scanning technology, while soft tissues have little effect. MRI has a high sensitivity to the imaging of soft tissues and blood vessels, and has little impact on bones. It can be seen that the morphological and functional information obtained by different imaging technologies in the same anatomy are different and complementary to each other. Therefore, proper integration of various imaging data has become an urgent problem for clinicians [19].

**Fig. 2:**
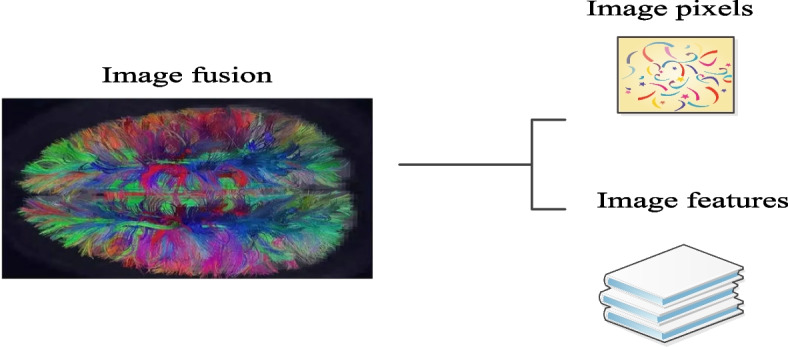
Way of image fusion technology

There are two kinds of existing image fusion technologies. One is based on image pixels, and the second is based on the characteristics of the image, as shown in Fig. 2:

#### Image pixels

Because pixels are the basic elements of an image, and the difference in gray values between pixels reflects the structural information contained in the image, a fused image can be obtained by calculating the weighted sum between the gray values of the pixels of two images, but this method has lost its original significance. The image fusion method based on pixel weighted average is relatively simple and fast, but in the fusion process, only the grayscale size of pixels is considered, ignoring the position of pixels and other factors. Therefore, the generated fusion image cannot well preserve the original image details, lose useful information, increase redundant information, and cause poor visual effects, making it difficult to distinguish the image.

The advantage of pixel level fusion is the process of directly fusing information from the pixels of an image. Limitations of pixel level fusion:

- 1. The scale of the original image data results in time-consuming algorithm implementation. Unprocessed data can overlap the advantages and disadvantages of the original sensor information, affecting the fusion effect;
- 2. The requirements for hardware facilities are quite high. When performing image fusion, the accuracy of registration is required to be accurate for each pixel between sensor data;
- 3. Due to pixel based calculations, pixel information is susceptible to pollution, noise, and other interference, resulting in unstable performance.

#### Image features

Feature level fusion is to extract feature information from an image and conduct comprehensive analysis and processing on it. The features obtained are sufficient expressions of pixels or sufficient statistics [20]. In the transformation area, it is necessary to determine specific feature selection criteria, and generate fusion images in the transformation area based on the selected rules. In principle, the inversion method is not easy and the algorithm is complex, but the actual application effect is better.

Its advantage is that the combination of original features forms features, increases the dimensionality of features, and improves the accuracy of the target. Feature vectors can be directly fused or recombined based on the attributes of the features themselves. Edges, shapes, and contour lights are important parameters for describing features, and their geometric transformations also have certain feature attributes. The features of an image are a form of cost processing that reduces the amount of data, preserves most of the information, and still loses some details.

**Fig. 3:**
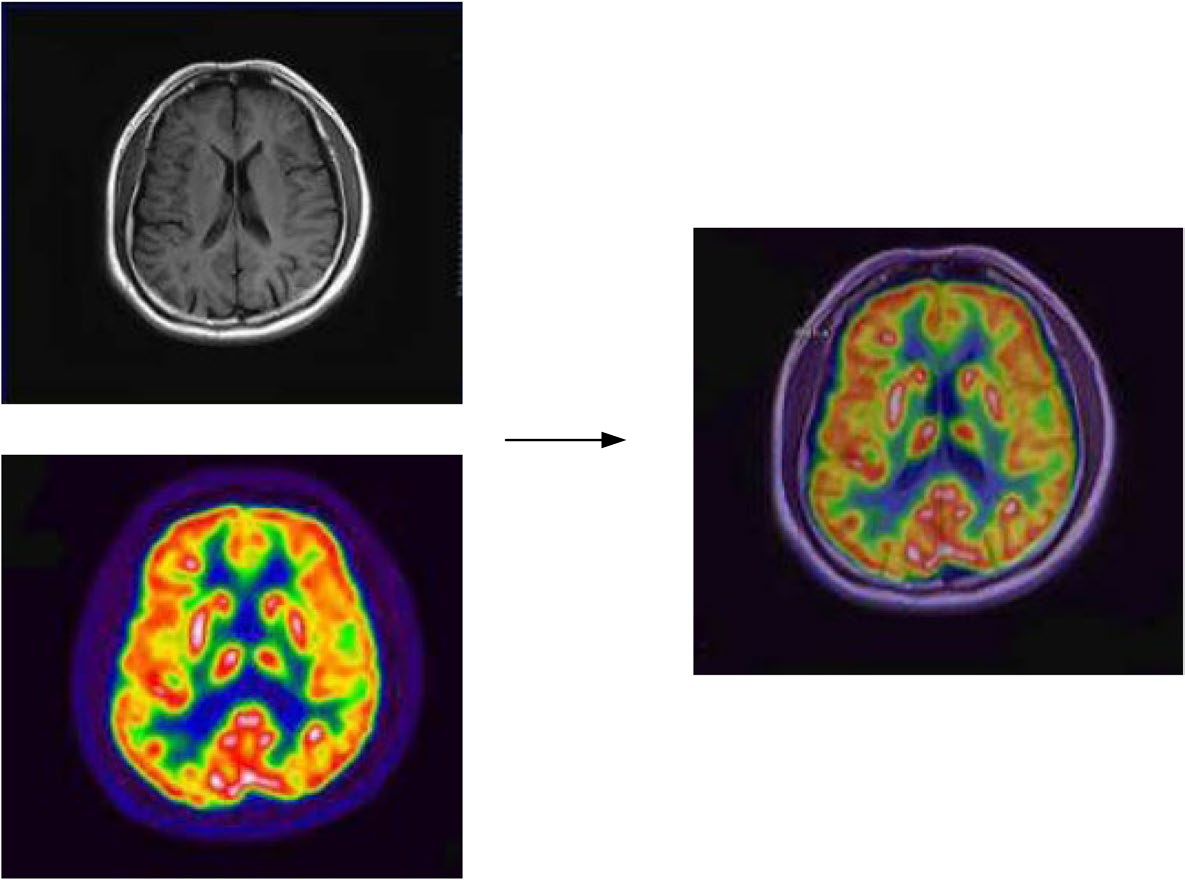
Images before and after fusion

The images before and after fusion are shown in Fig. 3.

### Machine learning-based medical imaging analysis in colorectal cancer health diagnosis and monitoring

Current colorectal cancer screening uses flexible colorectal toscopy, a procedure that involves endoscopic observation of the lining of the colon and rectal mucosa. The abnormal site is sliced for analysis, and although this is the standard diagnostic method, it also has drawbacks. First, the technology relies on visual detection, but it is difficult to detect small damage with the naked eye and often misses early malignant disease. Second, the endoscope can only detect changes in the intestinal surface and not the deep layers of the gut. Medical imaging research technology based on machine learning is based on optical coherence tomography. This technique enables high spatial and depth resolution at imaging depths of 1–2 mm. Examination normal and diseased tissues have different refractive indices and are very sensitive to morphological changes in early tumors. As technology advances, this technology can be used as a real-time, noninvasive imaging method to help with deeper cancer and early colorectal cancer screening.

## Factor of clinical application in diagnosis and monitoring of colorectal cancer

### Diagnosis of colorectal cancer

In case of any change in stool habits or bloody stool, the patient shall be promptly treated with digital rectal examination, X-ray contrast enema, sigmoidoscopy and fibrocolonoscopy [21]. X-ray barium double contrast can find the imaging features of intestinal wall defect, intestinal stenosis, mucosal destruction, etc., to determine the location and scope of the lesions. Sigmoidoscopy and fiber colonoscopy can directly observe the morphology of the entire colon mucosa. Biopsy of suspicious lesions helps to improve diagnostic accuracy, especially during the early detection of minor lesions. Rectal palpation is one of the simplest and most important examination methods to determine the tumor location, size, morphology, surgical approach, and prognosis. Many rectal cancer patients are often misdiagnosed as hemorrhoids and enteritis, leading to long-term treatment delay. Fecal occult blood detection is a simple and fast early diagnostic method. However, for patients with persistent and repeated occult blood positive and no cause, great attention should be paid to colon cancer, especially right colon cancer. Carcinoembryonic Antibody (CEA) is a disease related to malignant tumors but has no specificity, and can become an auxiliary examination method [22, 23].

### How to treat colorectal cancer

Although the vast majority of patients received surgical resection and adjuvant radiotherapy and chemotherapy, patients died due to various factors.

#### Surgical treatment

At present, radical treatment of colon cancer is still the first choice for surgical treatment. Most colorectal surgeons would recommend radical resection of invasive colorectal cancer, including complete resection of all masses visible and accessible to the naked eye during surgery, such as the primary focus and lymph nodes in the drainage area. Therefore, surgical treatment should be carried out for patients whose disease is limited to the primary or local lymph nodes, and also for patients with large local lesions that are difficult to completely remove after surgery, but have no distant metastasis. For patients with large local lesions that can be surgically removed but with distant metastasis, palliative resection can be performed to relieve obstruction and improve symptoms. For patients with extensive local lesions and fixed adhesions that cannot be removed, simple surgery or ostomy can be performed to alleviate the symptoms of the lesions. For patients with distant metastasis, palliative surgery can be performed according to the individual conditions of the patient if the primary lesion can still be removed.

After the operation for colorectal cancer, the intestinal motility is abnormal, leading to an increase in the number of stools. After sigmoidectomy, constipation is caused by the impairment of its function. The increase of stool frequency and fecal incontinence after anorectal and colonic anastomosis would cause the change of defecation function, and patients with rectal cancer would have the problem of urination function after operation. For patients without anus retention, a study is being conducted on the artificial anus placed in the perineum, as well as the equipment that can control the desire to defecate to solve the defecation problem of patients.

#### Radiotherapy

**Fig. 4:**
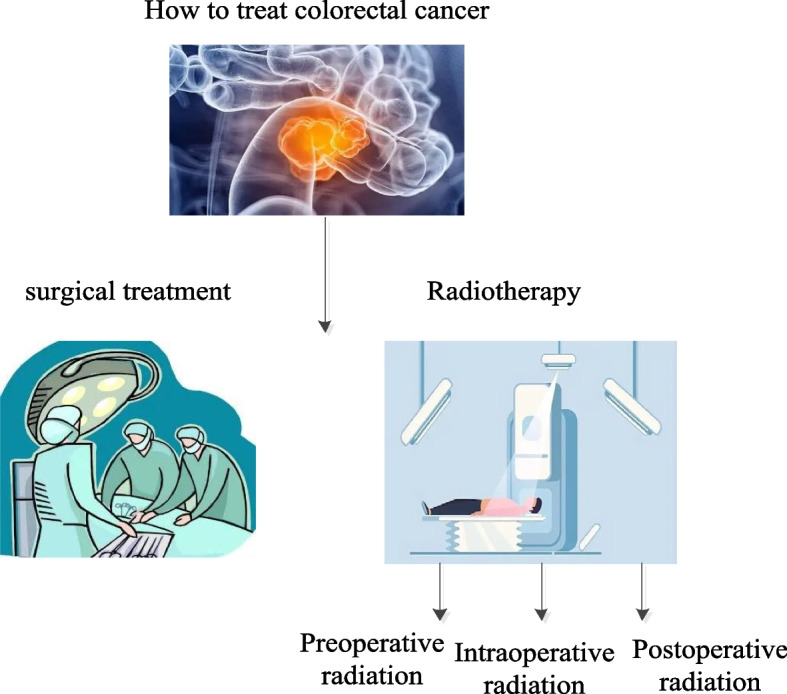
Schematic diagram of how the colorectal cancer is treated

The surgical cure rate and survival rate of colorectal cancer have been wandering for many years, and the reason for treatment failure is the high local recurrence rate, so comprehensive treatment is needed to improve the curative effect of colorectal cancer. At present, preoperative radiation, intraoperative radiation and postoperative radiation have been studied more and better in clinical practice. For patients with advanced rectal cancer with surgical contraindications, especially patients with local tumor invading adjacent tissues and with surgical contraindications, palliative radiotherapy can also achieve satisfactory results, as shown in Fig. 4 [24, 25].

## Medical image fusion algorithm model

### Low frequency coefficient fusion rule

Low frequency coefficients are used to represent the main information of the image. The neighborhood average method to obtain the fused low frequency coefficients can effectively improve the contrast and brightness of the image. First, the gray value of each 3 × 3 adjacent area is calculated and its maximum value is used as the low frequency fusion.

1 $$u\left(b\right)=\frac{1}{3\times 3}\sum_{n=0}^{1}\sum_{m=0}^{1}c(j+n,i+m)$$

2 $${L}_{k}^{z}\left(b\right)={L}_{y}^{X}\left(b\right),{L}_{k}^{X}(b)\ge {u}_{k}^{Y}(b)$$

3 $${L}_{k}^{z}\left(b\right)={L}_{k}^{Y}\left(b\right),{L}_{k}^{X}(b)<{u}_{k}^{Y}(b)$$

As shown in Formulas (1) and (2), $u(b)$ represents the average value of the neighborhood low-frequency coefficients centered on (i, j) in the sub image, and ${L}_{k\left(b\right),}^{X}{L}_{k\left(b\right),}^{Y}{L}_{k\left(b\right),}^{Z}$ represent the coefficients of NSCT decomposition of the source image X, Y and fusion image Z at layer K.

### High frequency coefficient fusion rule

The image in the image should include edges, lines and boundaries of the image, and be expressed by high-frequency subband coefficients. Because the three high-frequency signals decomposed by NSCT (Nonsubsampled Contourlet) have certain directionality, they can be divided into three different fine structures: horizontal, vertical and diagonal. Under the same decomposition scale, the coefficients of the corresponding regions of the three high-frequency sub regions are different, while the details of the image are dominated by high-frequency coefficients in the larger direction. Therefore, a large fusion criterion based on regional variance method is proposed, which not only takes into account the correlation between adjacent pixels, but also keeps the details of the original image as much as possible to ensure the image clarity, as shown in the following formulas:

4 $${\sigma }^{2}(b)=\frac{1}{3\times 3}\sum_{n=0}^{1}\sum_{m=0}^{1}[c\left(j+n\right),i+m-u\left(p\right)]^2$$

5 $${U}_{k}^{z}\left(b\right)={U}_{k}^{A}\left(b\right),{\sigma }_{k}^{A}(b)\ge {\sigma }_{k}^{B}(b)$$

6 $${U}_{k}^{z}\left(b\right)={U}_{k}^{B}\left(b\right),{\sigma }_{k}^{A}(b)<{\sigma }_{k}^{B}(b)$$

In the formulas, ${U}_{k}^{A}(b)$, ${U}_{k}^{B}(b)$, and ${U}_{k}^{Z}(b)$ respectively represent the *K* direction coefficients of source image *A*, *B*, and fusion image *Z* when the m-layer NSCT is decomposed.

### Structural similarity

Structural similarity is an index used to measure the similarity between images. Information such as image brightness *T*, contrast *D* and structure *J* are compared based on the visual characteristics of the human eye. It is defined as:

7 $$T(A,B)=\frac{2{\mu }_{A}{\mu }_{B }+{c}_{1}}{{\mu }_{A}^{2}+{\mu }_{B}^{2}+{c}_{1}}$$

8 $$D\left(A,B\right)=\frac{2{\sigma }_{A}{\sigma }_{B} +{c}_{2}}{{\sigma }_{A}^{2} +{\sigma }_{B}^{2}}$$

9 $$J\left(A,B\right)=\frac{{\sigma }_{AB }+{c}_{3}}{{\sigma }_{A}{\sigma }_{B} +{c}_{3}}$$

10 $$SSIM \left(A,B\right)=T \left(A,B\right)\cdot D \left(A,B\right)\cdot J (A,B)$$

Among them, the *A,B* in the figure are all input source images, and *T(A,B)* is the fusion result. *D(A,B)* is the source image; ${c}_{3}$ is the decomposed low-frequency coefficient, and *J(A,B)* is the decomposed high-frequency coefficient; $\sigma$ represents the low-frequency and high-frequency coefficients after fusion.

The following formula can also be further developed:

11 $$SSIM(A,B)=\frac{(2{\mu }_{A}{\mu }_{B} +{c}_{1})(2{\sigma }_{AB} +{ c}_{2})}{({\mu }_{A}^{2} +{ \mu }_{B}^{2}+{c}_{1})({\sigma }_{A}^{2} + {\sigma }_{B}^{2}+{c}_{2})}$$

Among them, AB is the image to be evaluated, and ${\mu }_{A}$ and ${\mu }_{B}$ are the average values of image AB; ${\sigma }_{A}^{2}$ and ${\sigma }_{B}^{2}$ represent the image AB, and ${\sigma }_{AB}$ represents the covariance of the image AB; ${c}_{1}$, ${c}_{2}$ and ${c}_{3}$ are constants.

## Overview of experimental materials and methods for clinical application in colorectal cancer diagnosis and monitoring

### General materials

Starting age for colorectal cancer (CRC) screening:

The paper suggested that clinicians offer CRC screening to all average-risk individuals age 45–49 (weak recommendation; low-quality evidence).

For average-risk individuals who have not initiated screening before age 50, it is recommended that clinicians offer CRC screening to all average-risk individuals beginning at age 50 (strong recommendation, high-quality evidence).

Colorectal cancer screening stop age:

The paper suggested that individuals who are up to date with screening and have negative prior screening tests, particularly high-quality colonoscopy, consider stopping screening at age 75 years or when life expectancy is less than 10 years (weak recommendation, low-quality evidence).

Therefore, the clinical data of 100 colorectal cancer patients admitted to a hospital in W region in 2017–2018 were selected. The samples were 60 men and 40 women, aged 45–65 years. The even number of samples stratified was used as the control group (group X, *n* = 50), and the odd number was used as the study group (group Y, *n* = 50). The male to female ratio of group X is 40:10; the age is 45–65 years old; the average age is (51.51 ± 4.65) years old; the male to female ratio of group Y is 39:11; the age is 51–76 years old; the average age is (52.56 ± 4.41) years old. There was no statistically significant difference in the overall data between group X and group Y.

### Treatment

Before the physical examination, patients underwent imaging with machine learning-based medical imaging AI,and before the physical examination, the doctor would let the patient take liquid food within two days, take magnesium sulfate and drink an appropriate amount of water before the physical examination, do not eat on the day of the physical examination and use normal saline for enema, and take an appropriate amount of hawthorn alkaloid 1 h before the physical examination. The routine examination of the patient was set by using the spiral CT scanner, and then the patient was placed in the supine position, from the diaphragm to the bottom of the pubic bone for routine examination.

### Observations

The fat cavity of the diseased intestinal wall is clear; the lateral side of the intestinal wall is smooth, and there is no protrusion node. Small patches can be seen in the fat space of the pathological intestinal wall in Phase 1 to 2, and the imaging image shows high density. In Phase 3 enhanced examination, the fat cavity in the intestine with protrusion node is not seen, and in Phase 4 enhanced scanning, the blurry boundary is displayed. Combined with imaging examination, the pathological grading was compared with that after operation.

### Statistical methods

SPSS18.0 software was used to make statistics on the collected data, and test counting was used. All measured data were expressed in *t*, and the test results showed that *P* < 0.05 had significant difference.

Two days before the physical examination, patients need to use machine learning based medical imaging for imaging. During this period, patients need to consume liquid food. It is necessary to consume an appropriate amount of magnesium sulfate within two days and take an appropriate amount of hawthorn alkaloids one hour before the physical examination. The patient needs to fast on the day of the physical examination and can only drink an appropriate amount of water. Before the physical examination, the patient needs to undergo physiological saline enema to ensure that the colorectal cavity is clearly visible. The set scanning parameters are used for abdominal scanning, covering the area from the diaphragm to the bottom of the pubic bone.

## Simulation experiment in colorectal cancer diagnosis and monitoring

### Staged preparation stage

According to the above stratified statistics, the patients were prepared by phases, as shown in Table 1.

**Table 1:** Preparation phase

group	Phase 1	Phase 2	Phase 3	Phase 4
Group X	11/11	6/13	5/12	7/14
Group Y	12/12	9/12	10/12	12/14
t	0	67.321	13.896	30.148
p	1	0	0	0

It can be seen from Table 1 that the Phases 2, 3 and 4 preparation stages of group Y were higher than those of group X and the difference was statistically significant.

### Comparison of pathological stages

In this study, all 100 patients were detected by machine learning as shown in Table 2. The diagnostic accuracy of medical image fusion in Phases 1–2 before clinical operation was 69.6%, 69.5% in Phase 3 and 69.4% in Phase 4.

**Table 2:** Comparison of clinical preoperative phase and postoperative pathological phase for medical image fusion diagnosis

Medical image fusion	Staging of disease			Total
	Phase 1–2	Phase 3	Phase 4	
Phase 1–2	11	7	0	23
Phase 3	5	38	6	41
Phase 4	0	14	19	36
Total	16	59	25	100

### The diagnostic rate analysis of medical imaging based AI diagnosis and traditional diagnosis

**Fig. 5:**
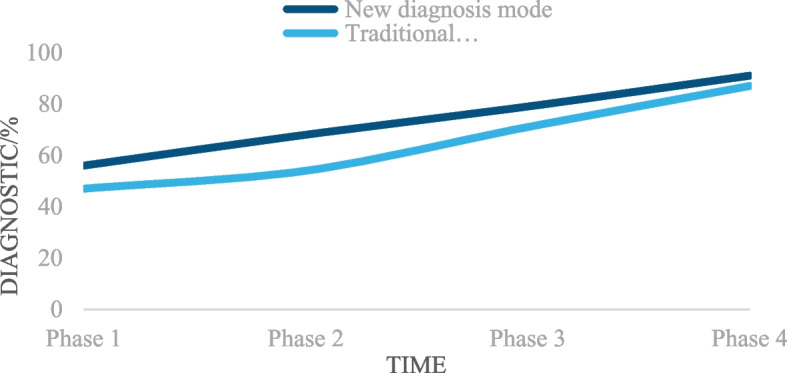
Medical image diagnosis and the diagnostic rate of traditional diagnosis

Because of the huge number of colorectal cancer patients and the imbalance of medical resources in various regions, it has not yet spread nationwide, so the diagnosis rate of colorectal cancer patients is very low. Figure 5 shows the diagnosis rate analysis of modal medical image fusion diagnosis and traditional diagnosis.

It can be seen from Fig. 5 that the curve trend of the broken line chart is that the curve trend of the new diagnosis mode and the curve trend of the traditional diagnosis mode are both gradually rising from Phase 1 to Phase 4. It can be seen that in the process of Phases 1–4, no matter which mode is used, and the diagnosis rate would rise steadily with the increase of time. The data analysis showed that the diagnostic rate of the new diagnosis method for Phase 1 patients was 56%, which is because patients would show various complications similar to colorectal cancer in the early stage, and the diagnostic rate of the new diagnosis method for Phase 2 patients was 68%. At this time, the diagnostic rate was higher than that of Phase 1 patients and there was time to deal with the disease; the diagnosis rate of the new diagnosis method for Phase 3 patients was 79%, and the treatment of the disease was more difficult. At this time, the diagnosis rate was much higher than that of Phase 1 and Phase 2; the diagnostic rate of the new diagnosis method for Phase 4 patients was 91%, and the diagnostic rate at this time can basically determine whether the patient has cancer. The diagnostic rate of the traditional diagnosis method for patients with Phases 1, 2, 3 and 4 was lower than that of the new diagnosis method, with the diagnostic rate of 47%, 54%, 71% and 87% respectively. The average diagnostic rate of the new diagnosis method in Phases 1–4 was 73.5%, while the average diagnostic rate of the traditional diagnosis method in Phases 1–4 was 64.75%. Through calculation, the average diagnostic rate of the new diagnosis method was 8.75% higher than that of the traditional diagnosis method.

### Analysis of the number of people who can effectively help the treatment process with multimodal medical image fusion diagnosis and traditional diagnosis

**Fig. 6:**
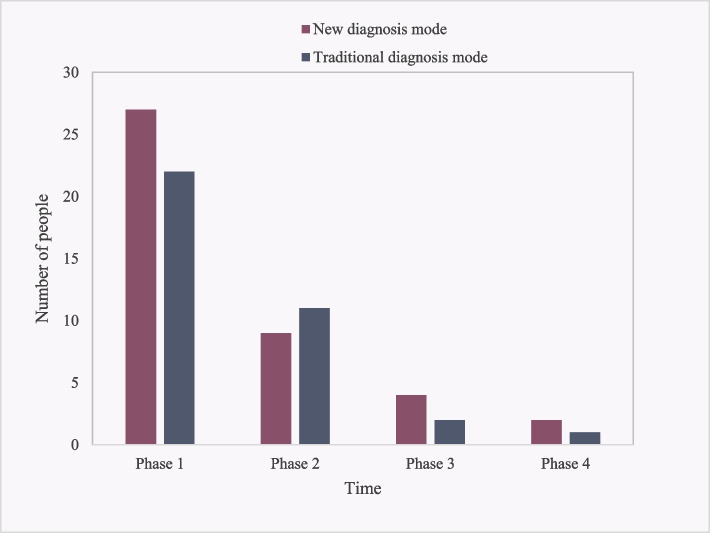
Treatment process helps with the effective numerical analysis

One hundred rectal cancer patients were equally divided into multimodal medical image fusion diagnosis and traditional diagnosis, and then the number of effective patients in the treatment process was statistically analyzed, as shown in Fig. 6:

It can be seen from Fig. 6 that the number of effective people of the new diagnosis method in the treatment process of Phases 1–4 was 42 and 84% of the total number, while the number of effective people of the traditional diagnosis method in the treatment process of Phases 1–4 was 36 and 72% of the total number. It can be calculated that the number of effective people of the new diagnosis method in the treatment process of colorectal cancer was 12% more than the number of effective people of the new diagnosis method. Among them, the number of effective patients in the Phases 1, 2, 3 and 4 of the new treatment was 27, 9, 4 and 2 respectively; the effective number of patients treated with traditional therapy in Phases 1, 2, 3 and 4 was 22, 11, 2 and 1, respectively. Therefore, it is inferred that the new diagnosis method based on multimodal medical image fusion is more effective than the traditional diagnosis method.

A comprehensive comparison was made between multimodal medical image fusion diagnosis, support vector machine, and random forest. Multimodal medical image fusion diagnosis performed better in the treatment of rectal cancer. Compared with support vector machines, multimodal medical image fusion diagnosis had significant advantages in accuracy and specificity. This method can more accurately distinguish different stages of lesions and improve the accuracy of diagnosis. This may be attributed to the comprehensive utilization of information from different medical imaging modalities by multimodal image fusion technology, providing a foundation for more comprehensive patient assessment. Compared to random forests, multimodal medical image fusion has achieved more significant gains in terms of effective number of people during the treatment process. This method not only outperforms in overall effectiveness, but also demonstrates better performance in various treatment stages, especially in the early stages. This indicates that multimodal medical image fusion diagnosis has significant advantages in early intervention of rectal cancer treatment.

### Analysis of satisfaction types of medical staff and patients with new diagnosis methods

**Fig. 7:**
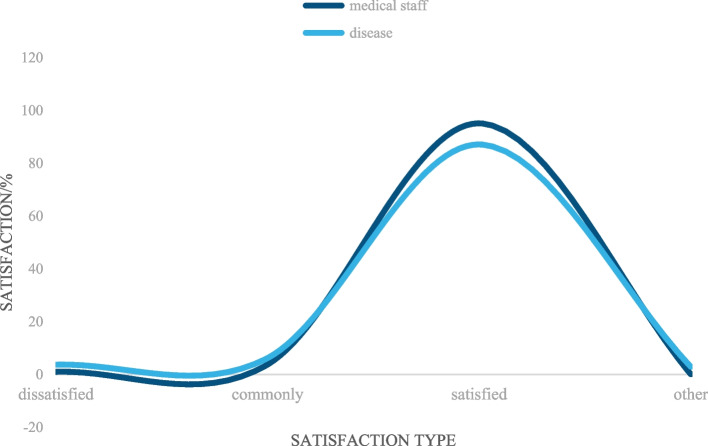
Analysis of the satisfaction types of medical staff and patients with the new diagnostic methods

Based on the above experimental research on colorectal cancer, the type analysis of satisfaction of medical staff and patients with the new diagnosis method is shown in Fig. 7:

It can be seen from Fig. 7 that the two curve trends of the broken line chart are that the curve trends of medical staff and patients both rise slowly from "dissatisfied" to "generally satisfied", then rapidly rise to "satisfied" and finally drop to "other". According to the curve trends, it can be seen that the satisfaction of medical staff and patients with the new diagnosis method was still high. Among the types of satisfaction of medical staff with the new diagnosis, the dissatisfaction rate was 1.01%; the general satisfaction rate was 3.78%; the satisfaction rate was 95.12%, and others were 0.09%; among the types of patients’ satisfaction with the new diagnosis, the dissatisfaction rate was 3.71%; the general satisfaction rate was 6.23%; the satisfaction rate was 87.12%, and others was 2.94%. It can be calculated that the satisfaction rate of medical staff was 8% higher than that of patients. Therefore, the clinical application of multimodal medical image fusion in colorectal cancer diagnosis and monitoring is worth promoting.

To sum up, machine learning-based medical imaging AI diagnosis is relatively effective in the diagnosis of colorectal cancer, and it is still worthy of clinical application and promotion for preoperative staging diagnosis and pathological staging results.

## Conclusions

At present, the incidence rate of rectal cancer in the elderly is very high, and the high incidence rate of men poses a threat to the lives of patients. Generally, it occurs in the middle and lower segment of the rectum and most of them have no symptoms. If it is diagnosed as the middle and late stage, it would have a great impact on the survival and prognosis of patients. If dysentery and enteritis are diagnosed according to the routine diagnosis, and the condition continues to deteriorate, including abdominal mass, intestinal obstruction and other symptoms, it is a relatively serious symptom. Further examination is required for persistent diarrhea, abdominal pain and other conditions. Preoperative staging treatment of rectal cancer can reduce postoperative complications and recurrence and improve the surgical effect.

Nowadays, with the continuous development and improvement of imaging diagnosis technology, the medical imaging AI diagnosis based on machine learning can effectively make up for the deficiency of other methods, so that rectal cancer patients can achieve relatively good treatment effects. It enables a more detailed description of the mass size, shape and relationship to the surrounding tissue, allowing clinicians to better understand the situation. It is important for patients to be fully prepared for multimodal medical image fusion scans to make the diagnosis more accurate and effective. Medical imaging of artificial intelligence based on machine learning diagnosis is based on the density of the surrounding tissue to distinguish different tissues. Because each layer of the rectum is soft tissue density of the difference is very small, radiology technology is used to identify whether violated the lower mucosa and muscle layer, which is not very good to distinguish between Phases 1 and 2. However, the article also have shortcoming in experiment. Given the more complex application scenarios of AI in the medical field, in order to achieve breakthroughs in medical AI from point to surface, it is necessary to have high-quality medical data support. Therefore, the training set database established in the early stage must be annotated and processed by experienced professional physicians. However, there are still differences in clinical experience among the physicians selected by various medical institutions for data annotation, resulting in uncertainty in the annotation results and subjective factors affecting the annotation results of the training dataset. At present, there is no strict and unified evaluation system and standards, making it difficult to ensure the effectiveness of each model. The use of medical imaging AI in the diagnosis of colorectal cancer is still affected by the quality of medical data and differences in annotation. In the future, a unified standard evaluation system would be established to improve the quality of medical data, standardize physician annotation, and ensure the consistency and reliability of model training.

In short, the medical imaging AI diagnosis based on machine learning has a good curative effect in the diagnosis of rectal cancer and has a good clinical application value in clinical practice.

## Data Availability

No datasets were generated or analysed during the current study.
